# Reliability and Validity of the CogState Battery Chinese Language Version in Schizophrenia

**DOI:** 10.1371/journal.pone.0074258

**Published:** 2013-09-02

**Authors:** Na Zhong, Haifeng Jiang, Jin Wu, Hong Chen, Shuxing Lin, Yan Zhao, Jiang Du, Xiancang Ma, Ce Chen, Chengge Gao, Kenji Hashimoto, Min Zhao

**Affiliations:** 1 Shanghai Mental Health Center, Shanghai Jiaotong University School of Medicine, Shanghai, China; 2 Division of Clinical Neuroscience, Chiba University Center for Forensic Mental Health, Chiba, Japan; 3 First Affiliated Hospital, Xi’an Jiaotong University Medical College, Xi’an, Shanxi, China; Rikagaku Kenkyūsho Brain Science Institute, Japan

## Abstract

**Background:**

Cognitive impairment in patients with schizophrenia is a core symptom of this disease. The computerized CogState Battery (CSB) has been used to detect seven of the most common cognitive domains in schizophrenia. The aim of this study was to examine the reliability and validity of the Chinese version of the CSB (CSB-C), in Chinese patients with schizophrenia.

**Methodology/Principal Findings:**

Sixty Chinese patients with schizophrenia and 58 age, sex, and education matched healthy controls were enrolled. All subjects completed the CSB-C and the Repeated Battery for the Assessment of Neuropsychological Status (RBANS). To examine the test-retest reliability of CSB-C, we tested 33 healthy controls twice, at a one month interval. The Cronbach α value of CSB-C in patients was 0.81. The test-retest correlation coefficients of the Two Back Task, Gronton Maze Learning Task, Social Emotional Cognition Task, and Continuous Paired Association Learning Task were between 0.39 and 0.62 (p<0.01) in healthy controls. The composite scores and all subscores for the CSB-C in patients were significantly (p<0.01) lower than those of healthy controls. Furthermore, composite scores for patients on the RBANS were also significantly lower than those of healthy controls. Interestingly, there was a positive correlation (r  =  0.544, p<0.001) between the composite scores on CSB-C and RBANS for patients. Additionally, in the attention and memory cognitive domains, corresponding subsets from the two batteries correlated significantly (p<0.05). Moreover, factor analysis showed a two-factor model, consisting of speed, memory and reasoning.

**Conclusions/Significance:**

The CSB-C shows good reliability and validity in measuring the broad cognitive domains of schizophrenia in affected Chinese patients. Therefore, the CSB-C can be used as a cognitive battery, to assess the therapeutic effects of potential cognitive-enhancing agents in this cohort.

## Introduction

Cognitive impairment is a characteristic feature at all stages of schizophrenia, affecting domains such as, attention, memory, executive function, and processing speed [1], [2]. Since this impairment is largely responsible for the loss of occupational, social and economic wellbeing [3], [4], restoring cognitive function is widely recognized as an important pharmacological treatment goal [5], [6]. For example, processing speed, one of the most severely impacted domain in patients with schizophrenia [7], correlates with daily life activities such as [3], job tenure [8] and independent living status [9]. Furthermore, a recent meta-analysis showed that cognitive impairment could be detected in subjects with prodromal psychosis [10]. For instance, children, who went on to develop schizophrenia in adulthood, displayed problems in verbal reasoning, working memory, processing speed and attention, compared with their peers [11]. Therefore, early detection of cognitive impairment in high-risk or prodromal subjects offers significant benefits for early therapeutic intervention in schizophrenia [12].

To date, there are few well accepted and comprehensive scales for measuring cognitive function of schizophrenia. The Measurement and Treatment Research to Improve Cognition in Schizophrenia (MATRICS) initiative-Consensus Cognitive Battery (MCCB) was developed in 2008, to measure cognitive function in patients with schizophrenia [13], [14]. This battery is approved by the Psychiatry Division of the Food and Drug Administration (FDA) in the USA, as the gold standard for the registration of trials targeting cognition in schizophrenia, and takes roughly two hours to complete by hand [15]. MCCB examines seven cognitive domains impaired in patients with schizophrenia, namely, processing speed, attention/vigilance, working memory, verbal learning, visual learning, reasoning and problem solving and social cognition [13]. However, a Chinese version of the MCCB is not yet available, although testing to validate the MCCB is currently underway.

The CogSate Battery (CSB), is a sensitive, computer based cognitive assessment instrument, suitable for schizophrenia patients aged between 6 and106 years [16]. It takes subjects approximately 40 minutes to complete. Previous studies show that the CSB is sensitive to cognitive impairment in schizophrenia, displaying good stability and reliability, and being largely free of practice effects on repeated applications [17], [18]. The CSB examines the seven cognitive domains found in the MCCB, and there is significant correlation between the subsets and composite scores of the two scales [19]. By contrast, the Chinese version of the Repeatable Battery for the Assessment of Neuropsychological Status (RBANS) [20] is typically used in Chinese patients with schizophrenia [21]–[24].

The aim of the current study was to investigate the reliability and validity of the Chinese language version of the CSB (CSB-C), in Chinese patients with schizophrenia. First, the test- retest reliability was assessed by examining inter-subset consistency in healthy controls. Second, criterion validity was examined by comparing composite scores and subsets from the CSB-C and RBANS in patients with schizophrenia and healthy controls. Third, construct validity to detect correlations between the subsets from CSB-C and RBANS in subjects was performed.

## Methods

### Subjects

Sixty hospitalized schizophrenia participants were recruited from Shanghai Mental Health center (SMHC), Shanghai, China. All patients met the Diagnostic and Statistical Manual of Mental Disorders criteria (DSM-IV) for schizophrenia [25]. Inclusion criteria included: (1) more than 9 years of education; (2) aged between 18 and 45 years of age; (3) Han nationality and proficient at Chinese; and (4) normal or corrected-to-normal vision and hearing. Exclusion criteria included: (1) any physical or neurological illness or trauma which could affect cognitive function (e.g., stroke, seizure, or severe head injury); (2) treatment with cognitive enhancing drugs within 6 months of study entry; and (3) substance dependence other than nicotine. There were no specific medication criteria for inclusion into the patient group. In addition, the Positive and Negative Syndrome Scale (PANSS) [26] was measured in patients with schizophrenia.

Of the recruited patients, 42 of 60 were treated with a single second-generation antipsychotic drug (risperidone, n = 14; olanzapine, n = 11, clozapine, n = 7; aripiprazole, n = 4; quetiapine, n = 3; paliperidone, n = 3; ziprasidone, n = 1), two patients were treated with chlorpromazine and 16 patients with a combination of antipsychotic drugs (olanzapine and haloperidol, n = 2; olanzapine and risperidone, n = 1; olanzapine and aripiprazole, n = 1; olanzapine and chlorpromazine, n = 1; clozapine and sulpiride, n = 1; clozapine and quetiapine, n = 1; clozapine and risperidone, n = 1; risperidone and aripiprazole, n = 1; risperidone and chlorpromazine, n = 1; risperidone and haloperidol, n = 1; haloperidol and quetiapine, n = 1; quetiapine and fluphenazine, n = 1; perphenazine and chlorpromazine, n = 1)

A total of 58 age, gender, and education duration matched healthy controls were recruited from the community. Healthy controls were required to be free of any Axis I disorders according to DSM-IV criteria. None had a first-degree family history of schizophrenia. And the inclusion and exclusion criteria were the same as for schizophrenic patients.

This study was approved by the Institutional Review Board (permission number: 2011-37R) of SMHC to ensure the highest standards of ethical consideration. Prior to commencement of the study, all subjects provided written, informed consent after receiving a full explanation of the study and any potential risks and benefits of study participation. The study was performed in accordance with the Declaration of Helsinki II.

### Instruments

The Chinese version of the Cognitive schizophrenia battery(CSB-C) contains eight tasks, including the Detection Task (DET, speed of processing), Identification Task (IDN, attention/vigilance), One Card Learning Task (OCL, visual learning and memory), Two Back Task (TWOB, working memory), International shopping List Task (ISLT, verbal learning and memory), the Groton Maze Learning Task (GML, problem solving/error monitoring), Social Emotional Cognition Task (SEC, social cognition), Continuous Paired Association Learning Task (CPAL, spatial working memory). These tasks are presented on a green screen, along with standardized instructions given by trained researchers before the commencement of each task, to ensure that subjects completely understood and followed the rules. Detailed procedures of the eight cognitive tasks have been described elsewhere [19]. The CSB-C results were uploaded to a secure account on the CogState server site (http://www.Cogstate.com), where data were calculated and normalization transformed (logarithmic transformation for reaction time, arcsine transformation for accuracy). The results of each domain on the CSB-C were calculated into Z-scores, where the healthy control mean was set to zero and the standard deviation to one, following the methodology used by Keefe et al [27]. A composite score was generated, with higher values representing better cognitive performance.

The Repeated Battery for the Assessment of Neuropsychological Status (RBANS) [20] is comprised of 12 subtests. These subsets are used to calculate five, age-adjusted index scores, including immediate memory (list and story memory task), visuospatial/constructional (figure copy and Line Orientation Task), language (Picture Naming and Semantic Fluency Tasks), Attention (Digit Span and Coding Tasks), and delayed memory (List Recall, Story Recall, Figure Recall, and List Recognition task). A higher RBANS score is associated with better cognitive function. The validity and reliability of the Chinese version of RBANS is used as a battery to detect cognitive impairment in patients with schizophrenia [28]–[30]. RBANS contains two versions for repeated measurement, and version A was used in this study.

The Social Adaptation Self-evaluation Scale (SASS) is a 21-item, self-reporting scale for evaluating broad areas of social functioning (e.g. spare time, work, family, life coping skills). Response scores range from 0 to 3 again, with higher scores representing better social adjustment [31]. The Chinese version of SASS shows good validity [32].

WHO-quality of life (QOL) instrument (WHOQOL-BREF) is a 26-item, self-administered questionnaire and is a shortened version of the WHOQOL-100 scale. It measures the four domains of physical health and wellbeing, psychological health and wellbeing, social relationships and environment, with higher scores representing a better quality of life [33]. The Chinese version of WHOQOL-BREF shows good reliability and validity [34].

### Procedures

During February 2012 to October 2012, 60 patients and 58 healthy controls were enrolled. Researchers collected demographic data and performed semi-structured interviews to obtain clinical histories. All subjects completed the CSB-C and RBANS in a quiet room. To prevent fatigue and withdrawal symptoms, subjects were allowed a short break of approximately 5 minutes or a cigarette. Subjects were also required to complete the self-administered SASS and WHOQOL-BREF. All participants completed the tests in their entirety. One month later, 33 healthy controls were retested using the CSB-C.

### Data Analysis

Statistical Product and Service Solutions (SPSS) was used for analysis. Continuous variables were examined by *t*-test and dichotomous variables were analyzed by the Chi-square test for group comparisons. Reliability analysis: Cronbach α value was calculated for internal consistency and Pearson correlation used for test-retest consistency. Discriminant validity: Z-score transformation was used to determine the magnitude of cognitive impairment in schizophrenia patients compared with healthy controls. Criteria validity: Pearson correlation analysis was used for relationships between the CSB-C and RBANS. Construct analysis: exploratory factor analysis was performed in schizophrenia patients. In the schizophrenia group, stepwise General Linear Models (GLM) with the CSB-C composite score as a dependent variable and with demographic data, illness duration, years of untreated psychosis, dosage of antipsychotic drug and PANSS scores as independent variables, were conducted to evaluate factors that impact on cognitive performance. Values of p<0.05 were considered statistically significant.

## Results

### Demographic and clinical data

There were no differences in demographic data between the two groups (**Table 1**). The illness duration of the patient group was 8.22 ± 5.7 years, and the total score on PANSS was 50.48 ± 8.92 year old (the mean ± SD). Patients showed significantly lower total scores on WHOQOL-BREF (*t*  =  –4.076, p<0.001) and its four subset scores (p<0.05) compared with healthy controls. By contrast, there were no differences in SASS scores between the two groups. This would indicate that unsurprisingly, patients with schizophrenia suffered a worse quality of life relative to healthy controls (**Table 1**).

**Table 1:** Demographic and clinical characteristic of the sample.

	Patients (n = 60)	Controls (n = 58)	t/x^2^	P
Age	31.47 (8.16)	30.83 (6.59)	–1.11	0.27
Years of education	12.37 (2.51)	13.91 (2.849)	0.47	0.64
Male, n (%)	33 (55.0%)	37 (63.8%)	0.95	0.33
Illness duration	8.22 (5.70)			
Years of untreated psychosis	1.36 (2.11)			
Chlorpromazine equivalents, mg/d	532.78 (368.72)			
PANSS positive	12.90 (3.44)			
PANSS negative	12.83 (4.45)			
PANSS total	50.48 (8.92)			
SASS	33.98 (7.62)	35.97(6.51)	–1.52	0.13
WHOQOL				
Physical health	24.03 (4.02)	26.40 (3.21)	–3.53	.001
Psychological health	19.70 (4.25)	21.67 (3.400)	–2.78	.006
Social relationship	9.17 (2.44)	11.60 (2.66)	–5.20	.000
Environment	25.48 (5.21)	28.52 (9.04)	–2.24	.027
Total score	77.55 (13.82)	86.77 (10.30)	–4.08	.000

The values are the mean (SD).

Student’s t-test and Chi-square test were used to examine differences between groups.

### Reliability of the CSB-C

All subjects completed the CSB-C battery within approximately 40 minutes. Administration time of the CSB-C was roughly 20 minutes longer than that of the RBANS.

In test-retest analysis, the subsets of TWOB, GML, SEC, SPAL showed significant correlation between test-retest performance, with correlation coefficients of 0.39 – 0.62 (p<0.05) (**Table 2**).

**Table 2:** Test-retest reliability of CSB-C in healthy subjects (n = 33).

CSB-C	r (test-retest)
DET	0.33
IDN	0.21
OCL	0.21
TWOB	0.49**
GML	0.62**
ISL	0.20
SEC	0.50**
CPAL	0.39*

* p<0.05, **p<0.01.

In patients, the Cronbach value of CSB-C was 0.81. The correlation coefficient of inter-subsets was 0.10–0.74, while that between composite score and subset was 0.58–0.80 **(** **Table 3** **)**.

**Table 3:** The correlation matrix of inter-subset for the schizophrenia patients.

Subsets	DET	IDN	OCL	TWOB	GML	ISL	SEC
IDN	0.64**						
OCL	0.36**	0.15					
TWOB	0.47**	0.30*	0.51**				
GML	0.33**	0.41**	0.42**	0.44**			
ISL	0.31*	0.47**	0.10	0.26*	0.20		
SEC	0.24*	0.15	0.37**	0.55**	0.37**	0.24*	
CPAL	0.26*	0.311*	0.39**	0.41**	0.74**	0.42**	0.46**
COMP	0.60**	0.61**	0.58**	0.71**	0.79**	0.58**	0.62**

* p<0.05,**p<0.01.

### Validity of the CSB-C

#### (1) Discriminant validity

**Figure 1:**
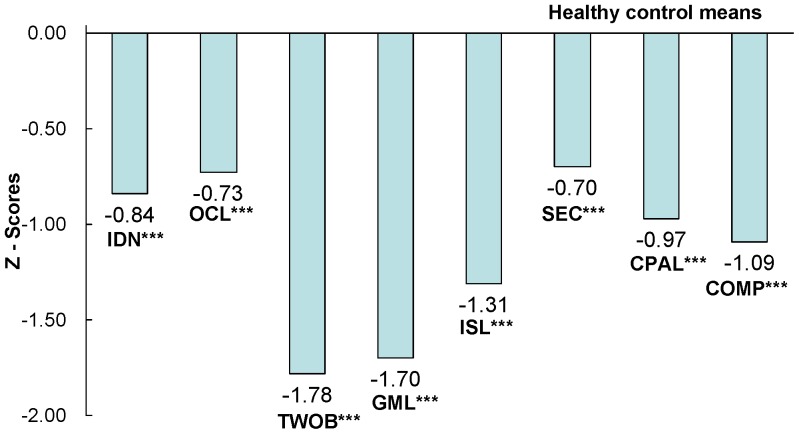
Magnitude of impairment relative to healthy controls in the CSB-C. Abbreviation: ISLT International Shopping List Task, DET Detection Task, IDN Identification Task, OCL One Card Learning Task, ONB One Back Task, CPAL Continuous Paired Association Task, GML Groton Maze Learning Task. Numbers of the figure are Z-score. **p<0.01, ***p<0.001.

**Figure 2:**
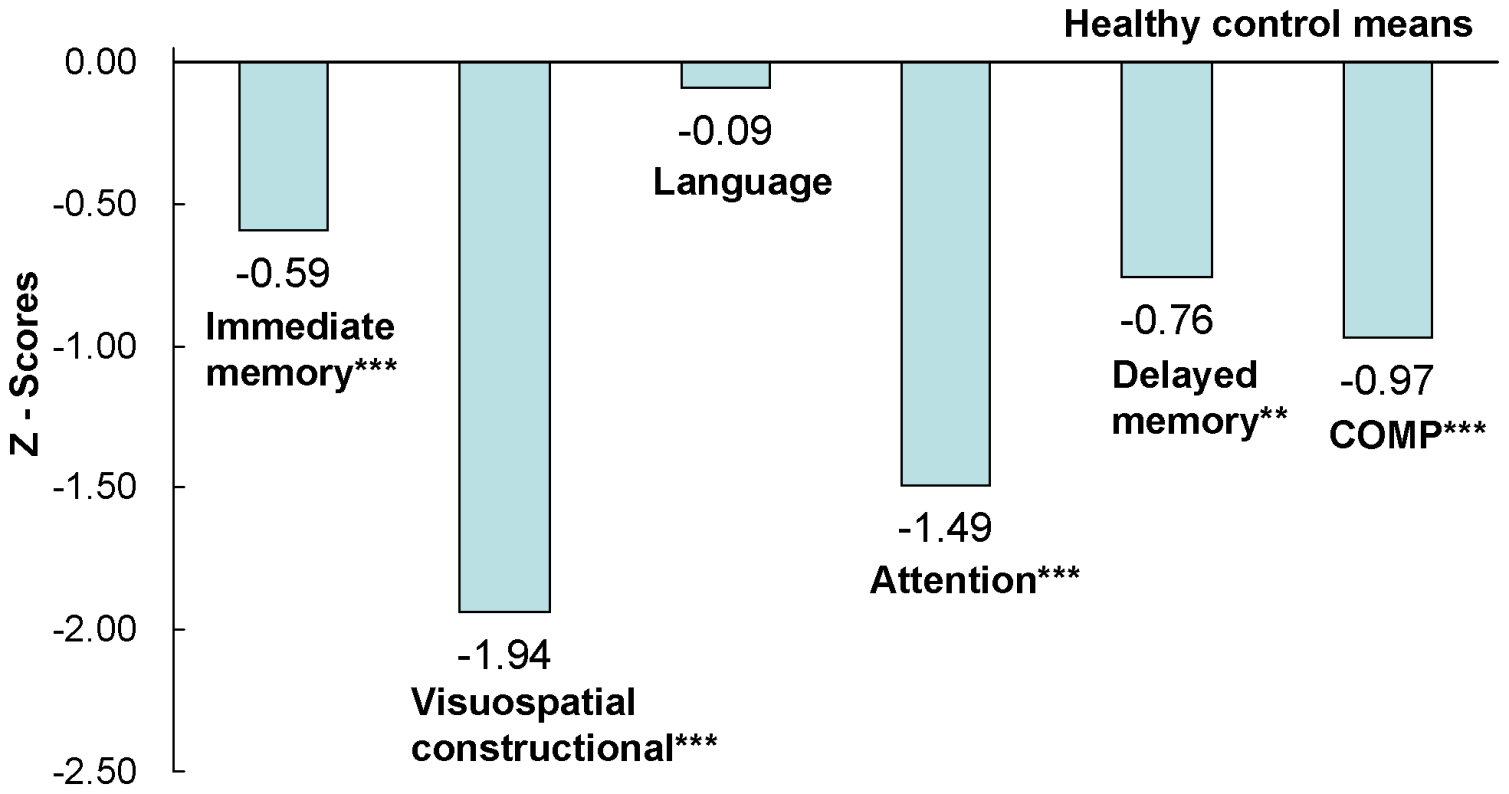
Magnitude of impairment relative to healthy controls in the RBANS. Numbers of the figure are Z-score. **p<0.01, ***p<0.001.

The performances of patients on the CSB-C and RBANS were significantly worse than those of healthy controls **(** **Figure 1** **,** **Figure 2** **)**, with the exception of the verbal subset in RBANS. Stepwise GLM showed that the duration of education could independently predict CSB-C composite scores. However, even after accounting for the variable of education, the differences between composite scores from both batteries remained significant (p<0.01).

#### (2) Criteria validity

**Figure 3:**
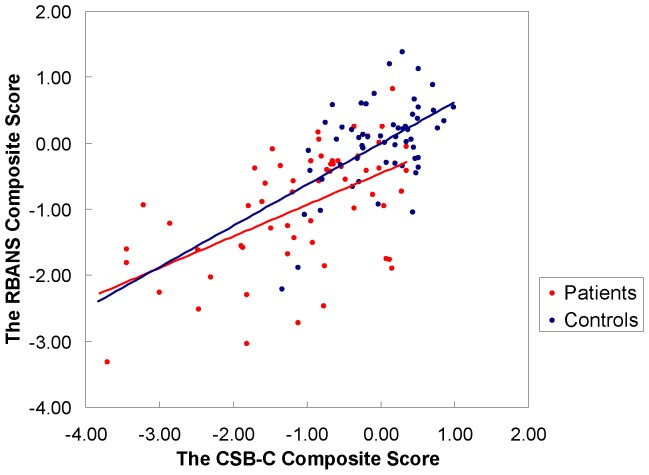
Inter-correlations between the CSB-C composite score and the RBANS composite score. Controls: r  = 0.53, p<0.01, Patients: r  =  0.544; p<0.001, Total subjects: r  =  0.67, p<0.001

The composite score of CSB-C correlated significantly with the equivalent score of RBANS (schizophrenia, r  =  0.54, p<0.001; total subjects, r  =  0.67, p<0.001) **(** **Figure 3** **)**. In patients, the cognitive domain of attention, measured by DET and IDN on CSB-C, significantly correlated with Attention on RBANS. In the memory cognitive domain, OCL, TWOB, GML and CPAL on the CSB-C significantly correlated with Immediate Memory, Delayed Memory, and Visuospatial/Constructional of the RBANS in patients. The SEC score on the CSB-C correlated significantly with all subscores of RBANS. However, there was no correlation between the ISL score on the CSB-C and any subset of RBANS **(** **Table 4** **)**.

**Table 4:** Correlation coefficients of CSB-C and RBANS in the schizophrenia group.

C R	Immediate memory	Visuospatial/constructional	Language	Attention	Delayed memory
DET	0.45**	0.16	0.07	0.27*	0.17
IDN	0.23	0.01	–0.11	0.28*	0.13
OCL	0.44**	0.38**	0.20	0.18	0.36**
TWOB	0.62**	0.40**	0.37**	0.34**	0.44**
GML	0.44**	0.21	0.17	0.14	0.45**
ISL	0.23	0.16	–0.09	0.22	0.16
SEC	0.54**	0.37**	0.32*	0.36**	0.51**
CPAL	0.37**	0.28*	0.12	0.18	0.36**

* p<0.05, **p<0.01.

#### (3) Construct validity

In patients, factor analysis was determined by adopting the Maximum Likelihood extraction methods, with oblique rotation. The Kaiser-Meyer-Olkin Measure (KMO) value for Sampling Adequacy was 0.705, and the chi-value of Bartlett’s Test of Sphericity, was 183.86 (p<0.001), which demonstrated common factors in the CSB-C, and therefore exploratory factor analysis was performed. The eigenvalue greater than one rule and scree plot converged in a two-factor solution that accounted for 60.92% of the total variance. Subsets that needed speed, such as DET and IDN were loaded on factor 1. Subsets that needed memory and executive function, such as OCL, TWOB, GML, CPAL, and ISL were loaded on factor 2. The SEC which asked for suitable reactions to social emotional expression, were also loaded on factor 2 **(** **Table 5** **).**

**Table 5:** Factor load of the CSB-C subsets in patients with schizophrenia.

Subsets (cognitive domains)	Factor 1	Factor 2
DET (processing speed)	**0.995**	–0.061
IDN (attention)	**0.589**	0.146
OCL (visual memory)	0.189	**0.401**
TWOB (working memory)	0.308	**0.401**
GML (error monitoring)	–0.027	**0.819**
ISL (verbal memory)	0.172	**0.344**
SEC (social emotional cognition)	0.015	**0.520**
CPAL (visual spatial working memory)	–0.185	**0.991**

## Discussion

To our best knowledge, this study is the first to report on the reliability and validity of the Chinese language version of the CSB in patients with schizophrenia. The Cronbach α value of CSB-C was 0.81 (Cronbach value > 0.8 suggestive of good reliability), which demonstrated that the CSB displayed good internal consistency in Chinese schizophrenics [35]. Furthermore, the test-retest reliability of healthy controls showed significant relationships in the subsets of TWOB, GML, SEC, and OCL, indicating good stability for the CSB-C, a finding consistent with previous studies [36], [37]. Falleti et al. [36] reported that the CSB showed good stability, and at an interval of one month, no practice effects in healthy adults. In addition, Hammers et al. [37] showed that CSB had acceptable stability and test-retest reliability with minimal practice effects for patients with dementia. Tasks in the CSB are presented by card, maze and pictures, and only the ISLT requires subjects to first hear and then recall as many words as possible, minimizing the impact of culture [38]. All subjects completed the test within approximately 40 minutes. Administration time of the CSB-C was roughly 20 minutes longer than that of the RBANS, however, the CSB-C assessed three additional cognitive domains. Taken together, these findings demonstrate that CSB-C is an acceptable battery to detect cognitive impairment in Chinese schizophrenics.

Performances on all the subset scores of CSB-C were significantly lower in schizophrenics, compared with healthy controls (about 1 SD magnitude), indicating that CSB-C is capable of detecting cognitive impairment in patients. These findings corroborate previous reports that several domains of cognitive impairment, including attention, learning and memory, executive function, speed of processing, working memory, and social cognition, persist in schizophrenia patients despite antipsychotic treatment [1], [2], [39]. It is this fact that makes the improvement of cognitive impairment a main therapeutic target in schizophrenia.

Composite scores on the CSB-C and RBANS showed significant correlations, even when data were adjusted for years of education. In the attention and speed of processing domain, DET and IDN subscores significantly correlated with Attention on RBANS. In the visuospatial memory and executive function domains, OCL, TWOB, GML, and CPAL, subscores also correlated significantly with Immediate Memory, Delayed Memory, and Visuospatial/Constructional (except for the GML score) in RBANS. In the social emotional cognitive domain, the SEC subscore correlated with all subscores on RBANS. These findings are consistent with previous studies [17], [19], [40]. In healthy adult subjects, CSB and MCCB showed moderate to large correlations, including the domains of processing speed, attention/vigilance, working memory, verbal and visual learning, reasoning/problem solving and social cognition [19]. This study found that social emotional cognition correlated with all subsets in RBANS, mainly because social emotional cognition is an integrated cognitive process, including facial expression memory and recognition, decision-making and problem solving [41]. However, we found no significant correlation between the ISL subset (verbal learning) of CSB-C and any subsets of RBANS. Immediate Memory on RBANS consists of List Learning and Story Memory. The possible reason for this, lack of correlation could be related to intact story memory function. However, we detected a trend for positive correlation of ISL on CSB-C and Immediate Memory on RBANS (r  =  0.227, p  =  0.09).

Factor analysis revealed two factors on CSB-C. The first, an attention and speed factor included DET and IDN, while the second, memory and reasoning/problem solving contained OCL, TWOB, GML, CPAL, ISL, and SEC. This is consistent with a previous study by Yoshida et al [40]. They performed factor analysis on CSB, in Japanese patients with schizophrenia and identified three factors, with the third factor separating out the SEC subset [40]. This suggests that social cognition may represent a separate cognitive domain in schizophrenia [40], [42]. As mentioned earlier, social cognition is an integrated process, related to encoding, storage, retrieval, reasoning, attention and memory [43]. Therefore, it is reasonable to categorize SEC as a memory and executive factor.

There are some limitations to this study. First, we compared a computerized cognitive test (CSB), against a conventional cognitive test (RBANS). Future research examining the validity of the CSB-C by comparing it with other computerized tests is required. Second, this study found no correlation between social emotional cognition and social adaptation. Since social cognition is an integrated process, assessment of social emotional perception recognition alone, will not provide a complete explanation for this complex process. Third, the sample size of this study was relatively small (n  =  60 for the patient group; n  =  58 for the control group). Further studies using larger sample sizes will be needed. Fourth, most patients enrolled in this study were medicated. It is well known that antipsychotic medication might affect cognition in patients with schizophrenia [39], [44]. Therefore, it is of interest to measure cognition in drug-naïve patients with schizophrenia or subjects with ultra-high risk.

Recently, Lieberman et al [45] reported that the α7 nicotinic receptor agonist, TC-5619, promoted a beneficial effect in the Groton maze Learning Task (GMLT: executive function) of CSB, in patients with schizophrenia. Therefore, CSB-C would be a useful cognitive battery to assess the therapeutic effects of cognitive enhancers in Chinese schizophrenics [45]. In addition, cognitive impairment has been shown in patients with other psychiatric diseases, including major depressive disorder [46]–[49], bipolar disorder [49]–[52], substance abuse [53], [54] and patients with cancer [55]–[57]. Therefore, CSB-C could also prove a useful tool in the measurement of cognitive impairment in these patients.

In conclusion, this study showed that CSB-C is sensitive to cognitive impairment in Chinese patients with schizophrenia, and that the CSB-C composite score correlated significantly with the composite score of RBANS Chinese language version. Therefore, CSB-C is a useful cognitive battery to assess the effects of cognitive enhancers in Chinese schizophrenics.

## References

[1] Keefe RS, Fox KH, Harvey PD, Cucchiaro J, Siu C, et al (2011) Characteristics of the MATRICS Consensus Cognitive Battery in a 29-site antipsychotic schizophrenia clinical trial. Schizophr Res 125: 161–168. 21075600 10.1016/j.schres.2010.09.015

[2] Wang CH, Li Y, Yang J, Su LY, Geng YG, et al (2013) A randomized controlled trial of olanzapine improving memory deficits in Han Chinese patients with first-episode schizophrenia. Schizophr Res 144: 129–135. 23352776 10.1016/j.schres.2012.12.021

[3] Evans JD, Heaton RK, Paulsen JS, Palmer BW, Patterson T, et al (2003) The relationship of neuropsychological abilities to specific domains of functional capacity in older schizophrenia patients. Biol Psychiatry 53: 422–430. 12614995 10.1016/s0006-3223(02)01476-2

[4] Mohamed S, Rosenheck R, Swartz M, Stroup S, Lieberman JA, et al (2008) Relationship of cognition and psychopathology to functional impairment in schizophrenia. Am J Psychiatry 165: 978–987. 18450928 10.1176/appi.ajp.2008.07111713

[5] Hyman SE, Fenton WS (2003) Medicine. What are the right targets for psychopharmacology? Science 299: 350–351. 12532001 10.1126/science.1077141

[6] Kaneko Y, Keshavan M (2012) Cognitive remediation in schizophrenia. Clin Psychopharmacol Neurosci 10: 125–135. 23430145 10.9758/cpn.2012.10.3.125 PMC3569160

[7] Dickinson D, Ramsey ME, Gold JM (2007) Overlooking the obvious: a meta-analytic comparison of digit symbol coding tasks and other cognitive measures in schizophrenia. Arch Gen Psychiatry 64: 532–542. 17485605 10.1001/archpsyc.64.5.532

[8] Gold JM, Goldberg RW, McNary SW, Dixon LB, Lehman AF (2002) Cognitive correlates of job tenure among patients with severe mental illness. Am J Psychiatry 159: 1395–1402. 12153834 10.1176/appi.ajp.159.8.1395

[9] Brekke JS, Long JD, Nesbitt N, Sobel E (1997) The impact of service characteristics on functional outcomes from community support programs for persons with schizophrenia: a growth curve analysis. J Consult Clin Psychol 65: 464–475. 9170770 10.1037//0022-006x.65.3.464

[10] Fusar-Poli P, Deste G, Smieskova R, Barlati S, Yung AR, et al (2012) Cognitive functioning in prodromal psychosis: a meta-analysis. Arch Gen Psychiatry 69: 562–571. 22664547 10.1001/archgenpsychiatry.2011.1592

[11] Reichenberg A, Caspi A, Harrington H, Houts R, Keefe RS, et al (2010) Static and dynamic cognitive deficits in childhood preceding adult schizophrenia: a 30-year study. Am J Psychiatry 167: 160–169. 20048021 10.1176/appi.ajp.2009.09040574 PMC3552325

[12] Sabbag R, Levin R, Edelman S, Heresco-Levy U (2011) Preventive pharmacological treatment --an evolving new concept in schizophrenia. Isr J Psychiatry Relat Sci 48: 82–90. 22120442

[13] Nuechterlein KH, Green MF, Kern RS, Baade LE, Barch DM, et al (2008) The MATRICS Consensus Cognitive Battery, part 1: test selection, reliability, and validity. Am J Psychiatry 165: 203–213. 18172019 10.1176/appi.ajp.2007.07010042

[14] Kern RS, Nuechterlein KH, Green MF, Baade LE, Fenton WS, et al (2008) The MATRICS Consensus Cognitive Battery, part 2: co-norming and standardization. Am J Psychiatry 165: 214–220. 18172018 10.1176/appi.ajp.2007.07010043

[15] Buchanan RW, Keefe RS, Umbricht D, Green MF, Laughren T, et al (2011) The FDA-NIMH-MATRICS guidelines for clinical trial design of cognitive-enhancing drugs: what do we know 5 years later? Schizophr Bull 37: 1209–1217. 20410237 10.1093/schbul/sbq038 PMC3196938

[16] Murthy NV, Mahncke H, Wexler BE, Maruff P, Inamdar A, et al (2012) Computerized cognitive remediation training for schizophrenia: an open label, multi-site, multinational methodology study. Schizophr Res 139: 87–91. 22342330 10.1016/j.schres.2012.01.042

[17] Maruff P, Thomas E, Cysique L, Brew B, Collie A, et al (2009) Validity of the CogState brief battery: relationship to standardized tests and sensitivity to cognitive impairment in mild traumatic brain injury, schizophrenia, and AIDS dementia complex. Arch Clin Neuropsychol 24: 165–178. 19395350 10.1093/arclin/acp010

[18] Pietrzak RH, Snyder PJ, Jackson CE, Olver J, Norman T, et al (2009) Stability of cognitive impairment in chronic schizophrenia over brief and intermediate re-test intervals. Hum Psychopharmacol 24: 113–121. 19090506 10.1002/hup.998

[19] Pietrzak RH, Olver J, Norman T, Piskulic D, Maruff P, et al (2009) A comparison of the CogState Schizophrenia Battery and the Measurement and Treatment Research to Improve Cognition in Schizophrenia (MATRICS) Battery in assessing cognitive impairment in chronic schizophrenia. J Clin Exp Neuropsychol 31: 848–859. 19142774 10.1080/13803390802592458

[20] Randolph C, Tierney MC, Mohr E, Chase TN (1998) The Repeatable Battery for the Assessment of Neuropsychological Status (RBANS): preliminary clinical validity. J Clin Exp Neuropsychol 20: 310–319. 9845158 10.1076/jcen.20.3.310.823

[21] Zhang XY, Liu L, Liu S, Hong X, Chen da C, et al (2012) Short-term tropisetron treatment and cognitive and P50 auditory gating deficits in schizophrenia. Am J Psychiatry 169: 974–981. 22952075 10.1176/appi.ajp.2012.11081289

[22] Wang JH, Li CB, Cheng Y, Yi ZH, Long B, et al (2009) Reliability and validity of repeatable battery for the assessment of neuropsychological status (RBANS) in schizophrenic patients: a preliminary study. Shanghai Archives of Psychiatry 21: 265–268.

[23] Zhang XY, Chen da C, Xiu MH, Haile CN, Sun H, et al (2012) Cigarette smoking and cognitive function in Chinese male schizophrenia: a case-control study. PLoS One 7: e36563. 22570726 10.1371/journal.pone.0036563 PMC3343009

[24] Zhang XY, Chen da C, Xiu MH, Yang FD, Tan YL, et al (2013) Thioredoxin, a novel oxidative stress marker and cognitive performance in chronic and medicated schizophrenia versus healthy controls. Schizophr Res 143: 301–306. 23238053 10.1016/j.schres.2012.11.017

[25] First MB, Spitzer L, Gibbon M, Williams JBW (1995) Structured clinical interview for DSM-IV axis I disorders. New York NY: Biometrics Research Department.

[26] Kay SR, Fiszbein A, Opler LA (1987) The positive and negative syndrome scale (PANSS) for schizophrenia. Schizophr Bull 13: 261–276. 3616518 10.1093/schbul/13.2.261

[27] Keefe RS, Goldberg TE, Harvey PD, Gold JM, Poe MP, et al (2004) The Brief Assessment of Cognition in Schizophrenia: reliability, sensitivity, and comparison with a standard neurocognitive battery. Schizophr Res 68: 283–297. 15099610 10.1016/j.schres.2003.09.011

[28] Wilk CM, Gold JM, Humber K, Dickerson F, Fenton WS, et al (2004) Brief cognitive assessment in schizophrenia: normative data for the Repeatable Battery for the Assessment of Neuropsychological Status. Schizophr Res 70: 175–186. 15329294 10.1016/j.schres.2003.10.009

[29] Azizian A, Yeghiyan M, Ishkhanyan B, Manukyan Y, Khandanyan L (2011) Clinical validity of the Repeatable Battery for the Assessment of Neuropsychological Status among patients with schizophrenia in the Republic of Armenia. Arch Clin Neuropsychol 26: 89–97. 21177761 10.1093/arclin/acq100

[30] Zhang BH, Tan YL, Zhang WF, Wang ZR, Yang GG, et al (2009) Repeatable Battery for the Assessment of Neuropsychological Status (RBANS) as a screening test in Chinese: reliability and validity. Chin Ment Heal J 28: 865–869.

[31] Bosc M, Dubini A, Polin V (1997) Development and validation of a social functioning scale, the Social Adaptation Self-evaluation Scale. Eur Neuropsychopharmacol 7 Suppl 1: S57–70; discussion S71–53. 10.1016/s0924-977x(97)00420-3 9169311

[32] Tse WS, Bond AJ (2007) Psychometric analysis of the Chinese version of Social Adaptation Self-evaluation Scale (C-SASS). Psychiatry Res 153: 277–281. 17688952 10.1016/j.psychres.2006.09.009

[33] Saxena S, Carlson D, Billington R (2001) The WHO quality of life assessment instrument (WHOQOL-Bref): the importance of its items for cross-cultural research. Qual Life Res 10: 711–721. 11871592 10.1023/a:1013867826835

[34] Xia P, Li N, Hau KT, Liu C, Lu Y (2012) Quality of life of Chinese urban community residents: a psychometric study of the mainland Chinese version of the WHOQOL-BREF. BMC Med Res Methodol 12: 37. 22452994 10.1186/1471-2288-12-37 PMC3364902

[35] Cortina JM (1993) What is coefficient alpha? An examination of theory and applications.. Journal of Applied Psychology 78: 98–104.

[36] Falleti MG, Maruff P, Collie A, Darby DG (2006) Practice effects associated with the repeated assessment of cognitive function using the CogState battery at 10-minute, one week and one month test-retest intervals. J Clin Exp Neuropsychol 28: 1095–1112. 16840238 10.1080/13803390500205718

[37] Hammers D, Spurgeon E, Ryan K, Persad C, Heidebrink J, et al. (2011) Reliability of repeated cognitive assessment of dementia using a brief computerized battery. Am J Alzheimers Dis Other Demen 26.. 10.1177/1533317511411907 PMC7469666 21636581

[38] Collie A, Darekar A, Weissgerber G, Toh MK, Snyder PJ, et al (2007) Cognitive testing in early-phase clinical trials: development of a rapid computerized test battery and application in a simulated Phase I study. Contemp Clin Trials 28: 391–400. 17267292 10.1016/j.cct.2006.10.010

[39] Keefe RS, Harvey PD (2012) Cognitive impairment in schizophrenia. Handb Exp Pharmacol: 11–37. 10.1007/978-3-642-25758-2_2 23027411

[40] Yoshida T, Suga M, Arima K, Muranaka Y, Tanaka T, et al (2011) Criterion and construct validity of the CogState Schizophrenia Battery in Japanese patients with schizophrenia. PLoS One 6: e20469. 21637776 10.1371/journal.pone.0020469 PMC3102733

[41] Rowland JE, Hamilton MK, Vella N, Lino BJ, Mitchell PB, et al.. (2012) Adaptive Associations between Social Cognition and Emotion Regulation are Absent in Schizophrenia and Bipolar Disorder. Front Psychol 3 : doi: 10.3389/fpsyg. 10.3389/fpsyg.2012.00607 PMC3573888 23423878

[42] Allen DN, Strauss GP, Donohue B, van Kammen DP (2007) Factor analytic support for social cognition as a separable cognitive domain in schizophrenia. Schizophr Res 93: 325–333. 17498927 10.1016/j.schres.2007.02.008

[43] Chung YS, Mathews JR, Barch DM (2011) The effect of context processing on different aspects of social cognition in schizophrenia. Schizophr Bull 37: 1048–1056. 20185539 10.1093/schbul/sbq012 PMC3160231

[44] Meltzer HY (2013) Update on typical and atypical antipsychotic drugs. Annu Rev Med 64: 393–406. 23020880 10.1146/annurev-med-050911-161504

[45] Lieberman JA, Dunbar G, Segreti AC, Girgis RR, Seoane F, et al (2013) A randomized exploratory trial of an alpha-7 nicotinic receptor agonist (TC-5619) for cognitive enhancement in schizophrenia. Neuropsychopharmacology 38: 968–975. 23303043 10.1038/npp.2012.259 PMC3629385

[46] Porter RJ, Gallagher P, Thompson JM, Young AH (2003) Neurocognitive impairment in drug-free patients with major depressive disorder. Br J Psychiatry 182: 214–220. 12611784 10.1192/bjp.182.3.214

[47] Hindmarch I, Hashimoto K (2010) Cognition and depression: the effects of fluvoxamine, a sigma-1 receptor agonist, reconsidered. Hum Psychopharmacol 25: 193–200. 20373470 10.1002/hup.1106

[48] Hasselbalch BJ, Knorr U, Kessing LV (2011) Cognitive impairment in the remitted state of unipolar depressive disorder: a systematic review. J Affect Disord 134: 20–31. 21163534 10.1016/j.jad.2010.11.011

[49] Yoshida T, Ishikawa M, Niitsu T, Nakazato M, Watanabe H, et al (2012) Decreased serum levels of mature brain-derived neurotrophic factor (BDNF), but not its precursor proBDNF, in patients with major depressive disorder. PLoS One 7: e42676. 22880079 10.1371/journal.pone.0042676 PMC3411809

[50] Harvey PD, Wingo AP, Burdick KE, Baldessarini RJ (2010) Cognition and disability in bipolar disorder: lessons from schizophrenia research. Bipolar Disord 12: 364–375. 20636633 10.1111/j.1399-5618.2010.00831.x

[51] Gigante AD, Bond DJ, Lafer B, Lam RW, Young LT, et al (2012) Brain glutamate levels measured by magnetic resonance spectroscopy in patients with bipolar disorder: a meta-analysis. Bipolar Disord 14: 478–487. 22834460 10.1111/j.1399-5618.2012.01033.x

[52] Bourne C, Aydemir O, Balanza-Martinez V, Bora E, Brissos S, et al.. (2013) Neuropsychological testing of cognitive impairment in euthymic bipolar disorder: an individual patient data meta-analysis. Acta Psychiatr Scand. 10.1111/acps.12133 23617548

[53] Dean AC, Groman SM, Morales AM, London ED (2013) An evaluation of the evidence that methamphetamine abuse causes cognitive decline in humans. Neuropsychopharmacology 38: 259–274. 22948978 10.1038/npp.2012.179 PMC3527116

[54] Baldacchino A, Balfour DJ, Passetti F, Humphris G, Matthews K (2012) Neuropsychological consequences of chronic opioid use: a quantitative review and meta-analysis. Neurosci Biobehav Rev 36: 2056–2068. 22771335 10.1016/j.neubiorev.2012.06.006

[55] Falleti MG, Sanfilippo A, Maruff P, Weih L, Phillips KA (2005) The nature and severity of cognitive impairment associated with adjuvant chemotherapy in women with breast cancer: a meta-analysis of the current literature. Brain Cogn 59: 60–70. 15975700 10.1016/j.bandc.2005.05.001

[56] Zachariae R, Mehlsen MY (2011) Is chemotherapy associated with cognitive impairment? Nat Rev Urol 8: 182–183. 21475329 10.1038/nrurol.2011.29

[57] Hodgson KD, Hutchinson AD, Wilson CJ, Nettelbeck T (2013) A meta-analysis of the effects of chemotherapy on cognition in patients with cancer. Cancer Treat Rev 39: 297–304. 23219452 10.1016/j.ctrv.2012.11.001

